# Supplementary material for: Causal Relationship between Aging and Anorexia Nervosa: A White-Matter-Microstructure-Mediated Mendelian Randomization Analysis
Source: Biomedicines. 2024 Aug 16;12(8):1874. doi: 10.3390/biomedicines12081874 (PMC11351342; doi:10.3390/biomedicines12081874)
Supplement: Supplementary file 1 [file biomedicines-12-01874-s001.zip › Supplementary Figure/Figure S9/Leaveoneout plot of MR analysis for ubm-b-1971 to AN.pdf]

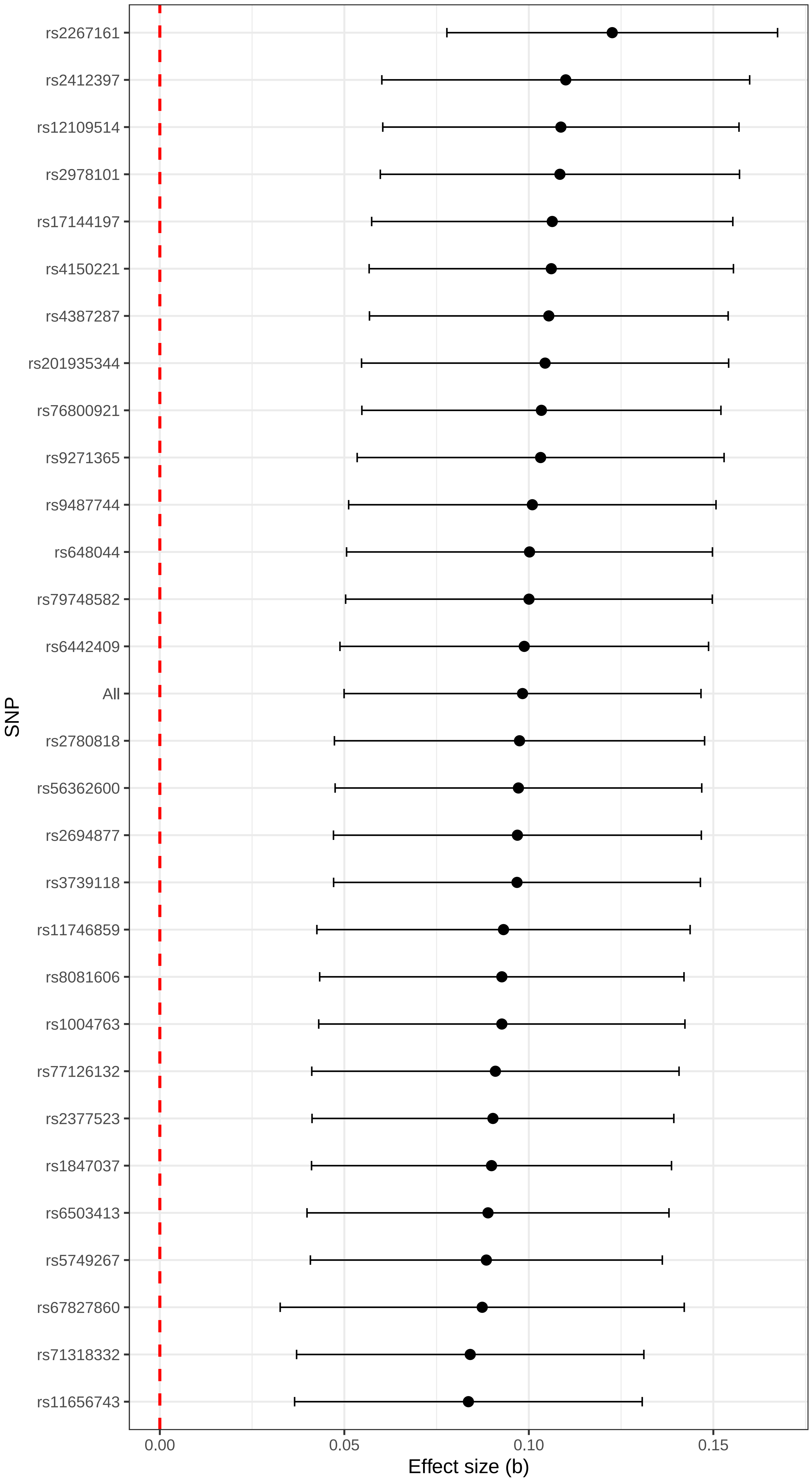

rs2267161
rs2412397
rs12109514
rs2978101
rs17144197
rs4150221
rs4387287
rs201935344
rs76800921
rs9271365
rs9487744
rs648044
rs79748582
rs6442409
All
rs2780818
rs56362600
rs2694877
rs3739118
rs11746859
rs8081606
rs1004763
rs77126132
rs2377523
rs1847037
rs6503413
rs5749267
rs67827860
rs71318332
rs11656743
SNP
0.00
0.05
0.10
0.15
Effect size (b)
